# Towards green biomanufacturing of high-value recombinant proteins using promising cell factory: *Chlamydomonas reinhardtii* chloroplast

**DOI:** 10.1186/s40643-022-00568-6

**Published:** 2022-08-13

**Authors:** Ke Ma, Lei Deng, Haizhen Wu, Jianhua Fan

**Affiliations:** 1 State Key Laboratory of Bioreactor Engineering, East China University of Science and Technology, 130 Meilong Road, Shanghai, 200237 People’s Republic of China; 2 Department of Applied Biology, East China University of Science and Technology, Shanghai, 200237 People’s Republic of China; 3 School of Chemistry and Chemical Engineering, Shihezi University, Shihezi, 832003 People’s Republic of China

**Keywords:** *Chlamydomonas reinhardtii*, Chloroplast, Synthetic biology, Green factory, Selectable marker, Protein expression

## Abstract

Microalgae are cosmopolitan organisms in nature with short life cycles, playing a tremendous role in reducing the pressure of industrial carbon emissions. Besides, microalgae have the unique advantages of being photoautotrophic and harboring both prokaryotic and eukaryotic expression systems, becoming a popular host for recombinant proteins. Currently, numerous advanced molecular tools related to microalgal transgenesis have been explored and established, especially for the model species *Chlamydomonas reinhardtii* (*C. reinhardtii* hereafter). The development of genetic tools and the emergence of new strategies further increase the feasibility of developing *C. reinhardtii* chloroplasts as green factories, and the strong genetic operability of *C. reinhardtii* endows it with enormous potential as a synthetic biology platform. At present, *C. reinhardtii* chloroplasts could successfully produce plenty of recombinant proteins, including antigens, antibodies, antimicrobial peptides, protein hormones and enzymes. However, additional techniques and toolkits for chloroplasts need to be developed to achieve efficient and markerless editing of plastid genomes. Mining novel genetic elements and selectable markers will be more intensively studied in the future, and more factors affecting protein expression are urged to be explored. This review focuses on the latest technological progress of selectable markers for *Chlamydomonas* chloroplast genetic engineering and the factors that affect the efficiency of chloroplast protein expression. Furthermore, urgent challenges and prospects for future development are pointed out.

## Introduction

**Fig. 1:**
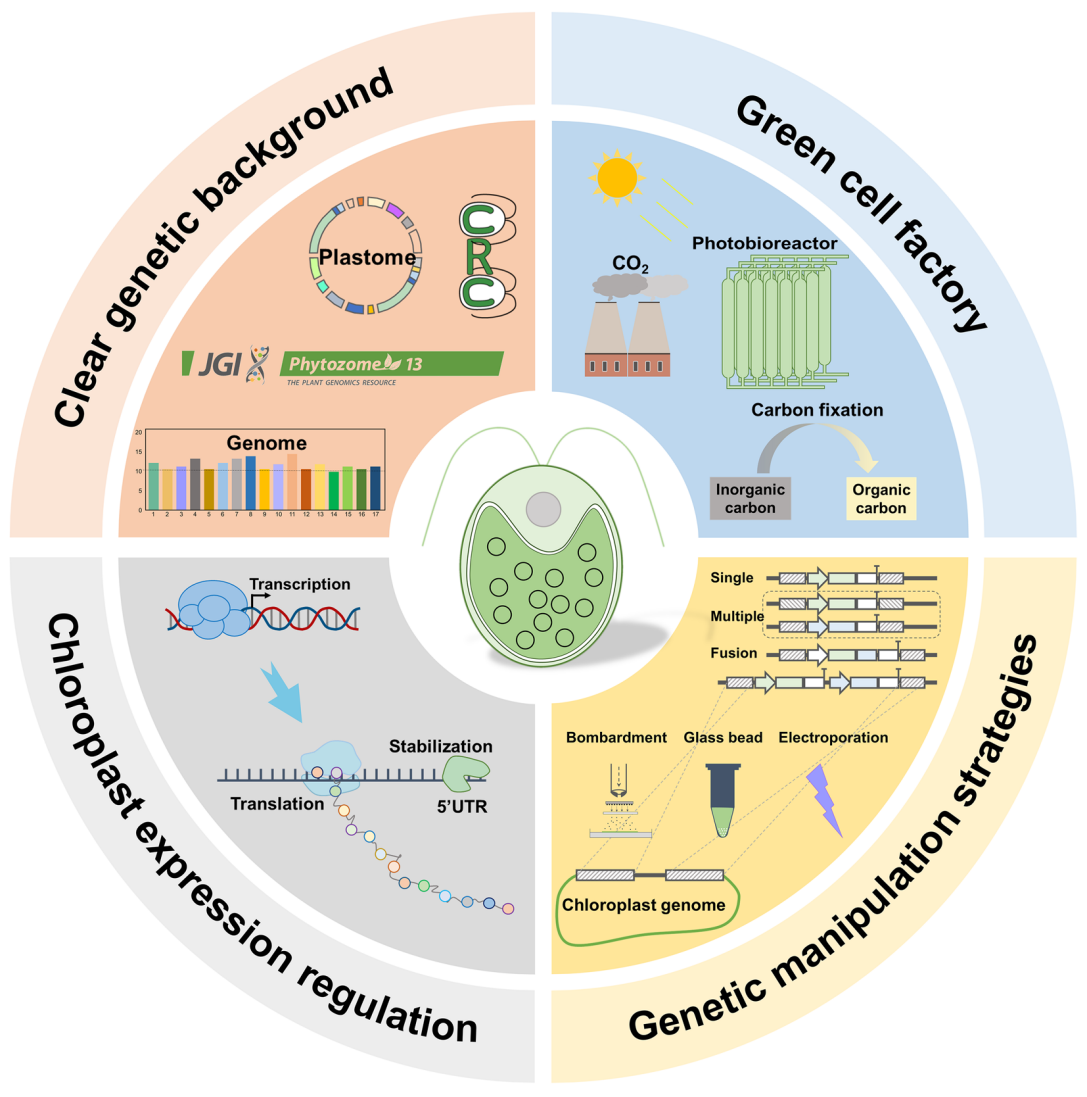
*Chlamydomonas* chloroplasts become an intriguing factory to manufacture the high-value products

Microalgae are superior photoautotrophic chassis organisms for synthetic biology, and have great potential for application in the direct fixation of CO_2_ to produce various bioenergy products (food, feed, enzymes, biochemicals, degradable plastics, biofuels, etc.) (Li et al. 2022). The large-scale industrialization of microalgae is in line with the carbon neutral strategy, helping to develop a green economy and promote sustainable development. As a model species of algae, *Chlamydomonas reinhardtii* is generally recognized as safe (GRAS) and has been employed to produce bioenergy products through metabolic engineering methods (Kato et al. 2022). *Chlamydomonas* has a well-defined genetic background and is amenable to multiple types of transgenic manipulations. The availability of a near-complete mutant library is also one of the unique advantages of *C. reinhardtii* (Li et al. 2019; Fauser et al. 2022)*.* These *Chlamydomonas* mutants have important implications for research in the fields of basic biology, global carbon fixation and synthetic biology. Besides, *Chlamydomonas* chloroplasts have become a unique natural factory for the production of high-value exogenous proteins due to their prokaryotic expression characteristics, high-copy plastid genome, and compatibility with post-translational modifications (Ahmad et al. 2020) (Fig. 1).

The chloroplast genome of *C. reinhardtii* was first assembled in 2002 (Maul et al. 2002). The newest assembly shows that *Chlamydomonas* chloroplast is a circular genome of 205, 535 bp, containing two single-copy regions separated by two large inverted repeat sequences (~ 22 kb). The plastome encodes 108 genes (including rRNA, tRNAs and protein-coding genes) (Gallaher et al. 2018). The review has revealed that the foreign DNA insertion into plastids occurs exclusively through homologous recombination, which allows transgenes to enter specific loci with precision and predictability (Esland et al. 2018). Because of the apparent lack of gene silencing mechanism in chloroplasts, high levels of transgene expression are achieved by fusing the transgene to cis-elements (promoters and untranslated regions) and transgene expression remains stable in the absence of selection pressure. The product can be further modified simply after translation, such as disulfide bond formation (Mayfield et al. 2003). Combined with advances in synthetic biotechnology development, *Chlamydomonas* chloroplasts can serve as a testbed for synthetic biology (Jackson et al. 2021; Cutolo et al. 2022).

More than 100 different foreign proteins have been successfully produced in the *Chlamydomonas* chloroplast (Jackson et al. 2021), most of which were introduced into the plastome via a single cassette containing both the gene of interest and a resistance selectable marker. In addition, multigenic engineering was achieved, up to six gene cassettes in parallel (Gimpel et al. 2016; Macedo-Osorio et al. 2018; Larrea-Alvarez and Purton 2020; Jackson et al. 2022). Due to the limited diversity of dominant resistance selectable markers and the risk of horizontal transfer to other environmental organisms of resistance genes, the notable bottlenecks in genetic engineering are development of new dominant markers or recycling of resistance markers. Notably, strategies to cope with these challenges have been proposed or development in recent reports, as discussed later in this review (Sandoval-Vargas et al. 2018, 2019; Changko et al. 2020).

Chloroplast gene expression is a complex process that could be affected at the level of transcription, post-transcriptional mRNA processing, mRNA stability, translation initiation, protein stability, and protein transport (Eberhard et al. 2002; Marín-Navarro et al. 2007; Stern et al. 2010). Moreover, many of these processes are regulated by nuclear-encoded factors, which are usually gene-specific. Some of the factors were employed as tools to enhance the expression of the protein of interest (Surzycki et al. 2007; Carrera-Pacheco et al. 2020; Shahar et al. 2021).

In this paper, the current status of *Chlamydomonas* chloroplast gene engineering is summarized, and common strategies for heterologous genes expression using the *Chlamydomonas* chloroplast as an expression factory are discussed. Factors affecting gene expression are classified and summarized. Furthermore, perspectives on development of new genetic tools for chloroplast gene engineering in the future are provided.

## The urgent need for green sustainable cell factories

*Chlorophyta* have been subdivided into four main classes (*Chlorophyceae*, *Prasinophyceae*, *Trebouxiophyceae* and *Ulvophyceae*) (Leliaert et al. 2012). The unicellular green alga *C. reinhardtii* (belongs to *Volvocales* from *Chlorophyceae*) has a well-defined genetic background, can be genetically manipulated, and has been used as a model organism in multiple different research fields over the past few decades (Salomé and Merchant 2019). *Chlamydomonas* is generally recognized as a safe host for the production of biofuels and high-value recombinant products, and has been successfully employed as a green cell factory for the production of various recombinant proteins, including antibodies, immunotoxins, hormones, protein drugs, vaccines, and industrial enzymes (Dyo and Purton 2018). However, transgene incorporation into the *C. reinhardtii* nuclear genome is a complex process. Transgenes targeting the nucleus are often randomly truncated by endogenous nucleases, and exogenous genes successfully integrated into the nuclear genome are likely to be difficult to express along with gene silencing (Tran and Kaldenhoff 2020). In contrast to the low yields achieved by nuclear-expressed transgenes of *C. reinhardtii*, the levels of heterologous proteins from chloroplast transgenic production were generally much higher. Chloroplasts of *C. reinhardtii* support efficient accumulation of recombinant proteins that are properly folded into biologically active soluble proteins. *Chlamydomonas* chloroplast is a suitable expression platform, with reported yields ranging from 0.5 to 5% of total soluble protein (TSP) (Taunt et al. 2018).

*Chlamydomonas* chloroplast expression products can be divided into immunogens, antibodies, functional drugs, food additives, etc. according to the application classification (Table 1). However, hitherto products based on recombinant expression of *C. reinhardtii* have not been used for commercial large-scale production. Recently, the National Health Commission of the People’s Republic of China approved *C. reinhardtii* as a new food raw material (NHC 2022). Globally, the existing microalgae companies have only developed some primary products, such as microalgae powder, natural oils (especially polyunsaturated fatty acids EPA and DHA), pigments (such as astaxanthin and phycocyanin), etc. These products are used in the fields of food, feed, pharmaceuticals, cosmetics and biofuels (Hachicha et al. 2022).

**Table 1:** Recombinant protein expressed in *C. reinhardtii* chloroplast (the case with the highest expression in each application category)

Protein	Category	Description	Accumulation	References
VP28	Antigen	The envelope protein of shrimp white spot syndrome virus (WSSV)	20.9% TCP	Surzycki et al. 2009
HMGB1	Damage-associated molecular patterns	High mobility group protein 1, mediating damage repair, recruiting innate immune cells and inducing inflammatory cytokine expression	2.5% TSP	Rasala et al. 2010
Pal/Cpl1	Antimicrobial protein	Endolysin, specifically kills the human pathogen *Streptococcus* *pneumoniae*	1.2% TSP	Stoffels et al. 2017
14FN3	Antibody mimic	Human fibronectin type III (FN3) domain 14	3% TSP	Rasala et al. 2010
V_H_H	Nanobody	V_H_H domains derived from alpacas, acting as an antitoxin to neutralize toxins produced by toxic microorganisms	4.6% TSP	Barrera et al. 2015
M-SAA	Therapeutic protein	Bovine milk isoforms of serum amyloid A, stimulating intestinal epithelial cells to secrete mucin	5% TSP	Manuell et al. 2007
VEGF	Growth factor	Vascular endothelial growth factor, promoting the growth of vascular endothelial cells and inducing vascular proliferation	2% TSP	Rasala et al. 2010
hGh	Protein hormone	Human growth hormone, promoting human growth and development	0.5 mg/L culture	Wannathong et al. 2016

*TSP* total soluble protein; *TCP* total cell protein

With the advancement of sequencing and omics technologies, a growing amount of green microalgal chloroplast genome information was revealed in addition to that of *Chlamydomonas* (Table 2). These data indicate that there will be numerous algal chloroplasts that may be developed and utilized in the future to express a variety of proteins. Furthermore, development of genetic engineering inspires commercial application of eukaryotic microalgae (Shi et al. 2021).

**Table 2:** Representative chloroplast genome information (*Chlorophyta*)

Class	Species	Year	Size (kb)	Gene number	GC content (%)	References
*Chlorophyceae*	*Chlamydomonas reinhardtii*	2002	205.5	108	34.6	Maul et al. 2002; Gallaher et al. 2018
	*Scenedesmus obliquus*	2006	161.5	96	26.9	de Cambiaire et al. 2006
	*Dunaliella salina*	2010	269.0	102	32.1	Smith et al. 2010
	*Haematococcus pluvialis*	2021	1351.6	83	50.1	Ren et al. 2021
*Prasinophyceae*	*Monomastix* sp. OKE-1	2009	114.5	94	39.0	Turmel et al. 2009
	*Nephroselmis olivacea*	1999	200.8	127	42.1	Turmel et al. 1999
	*Ostreococcus tauri*	2007	71.7	86	39.9	Robbens et al. 2007
	*Pyramimonas parkeae*	2009	101.6	110	34.7	Turmel et al. 2009
	*Pycnococcus provasolii*	2009	80.2	98	39.5	Turmel et al. 2009
*Trebouxiohyceae*	*Chlorella vulgaris*	1997	150.6	112	31.6	Wakasugi et al. 1997
	*Parachlorella kessleri*	2009	124.0	112	30.0	Turmel et al. 2009
	*Trebouxia* sp. Tr9	2019	303.2	138	31.9	Martínez-Alberola et al. 2020
*Ulvophyceae*	*Oltmannsiellopsis viridis*	2006	151.9	105	40.5	Pombert et al. 2006
	*Pseudendoclonium* *akinetum*	2006	195.9	108	31.5	Pombert et al. 2006
	*Pedinomonas minor*	2009	98.3	105	34.8	Turmel et al. 2009

## High-efficiency gene delivery strategy

The most common method for transgenic *C. reinhardtii* chloroplasts is microparticle bombardment. Driven by high-pressure helium, DNA-coated gold or tungsten particles bombard solid plates coated with microalgal cells. Although particle bombardment produces stable conversions, the bombardment equipment and the gold or tungsten particles are expensive (Shamriz and Ofoghi 2016). More economical methods are electroporation and vortexing in the presence of glass beads. In the former method, cracks appeared in the cell and chloroplast membrane structure after electric shock, while in the latter, due to the abrasive action of glass beads, pores were generated in the cell and chloroplast membrane, and DNA directly enters the chloroplast. However, electroporation transformation of chloroplasts is rarely reported, and glass bead vortexing also requires consideration of cell type. The removal of cell wall might improve the transformation efficiency (Hwang et al. 2018). Transformation efficiencies of electroporation, glass bead vortexing and microparticle bombardment are shown in Table 3. Notably, there are multiple copies of the genome in *Chlamydomonas* chloroplasts, requiring multiple rounds of homologous recombination to get the transgene into each copy. This is why multiple rounds of selection are required to yield stable, homoplasmic clones.

**Table 3:** Comparison of electroporation, glass bead vortexing and microparticle bombardment for Chlamydomonas chloroplast transformation

Transformation methods	Pre-treatment	Complexity	Transformation efficiency	Cost	References
Electroporation	Cells premixed with sucrose solution	**++**	Unknown	**++**	Yakun et al. 2006
Glass bead	Removal of cell wall	**+**	50 clones/μg DNA	**+**	Kindle et al. 1991
Bombardment	Precipitation of DNA onto microcarriers	**+++**	40–100 clones/μg DNA	**+++**	Lee et al. 1998

Most of the existing cases of successful expression of chloroplast transgenes in *Chlamydomonas* are transformation of a single exogenous gene. There are many ways to achieve the transformation of multiple foreign genes. For example, co-transformation using multiple vectors allows multiple expression cassettes to be independently integrated into different loci in the genome (Larrea-Alvarez and Purton 2020). However, this method leads to difficulties in transformant screening, and often requires the use of multiple selectable markers, whereas too many resistance genes as selection markers are not popular in practical applications. Another method to transform multiple foreign genes is to ligate two or more expression cassettes into the same vector. This way is more commonly used because only a single screening marker needs to be carried. This method is limited by the maximum load that the vector able to carry. Therefore, linkers can be added between the coding regions of different exogenous genes to achieve multi-gene expression. Macedo-Osorio et al. used the endogenous intercistronic region of *Chlamydomonas* as a linker to connect the antibiotic marker gene and green fluorescent protein, and the bicistronic successfully expressed mRNA and protein (Macedo-Osorio et al. 2018). This linker has also been successfully used for fusion expression of dual marker proteins (Jackson et al. 2022). In nuclear genome editing, the 2A peptide gene from foot and mouth disease virus (FMDV) was shown to be successfully used for the fusion expression of two genes. The viral 2A peptide can achieve separate expression of the two proteins during translation through a mechanism of ribosome skipping (Rasala et al. 2012). This linker peptide might be used in chloroplast gene expression in the future.

## Existing resistance markers

Since the expression mechanism in *C. reinhardtii* chloroplast is similar to that in prokaryotes, some antibiotics act on prokaryotic ribosome complex may also act on ribosome of chloroplast. Whereas there are only two antibiotic resistance genes were successfully used as dominant selectable marker. Besides, mutations in ribosomal proteins or in rRNA can confer resistance to several antibiotics.

### aadA

The *aadA* sequence that encoded aminoglycoside 3′ adenosine transferase derived by *Escherichia coli* (Hollingshead and Vapnek 1985), was introduced into *Chlamydomonas reinhardtii* chloroplast genome through microprojectile bombardment. The exogenous gene was stable in chloroplast and its encoded product conferred resistance to spectinomycin and streptomycin in transformed cells (Goldschmidt-Clermont 1991), which become the first successful example of the stable expression of a foreign protein in a transgenic chloroplast. The *aadA* gene is now a portable selection marker widely used in chloroplast transformation. This gene can be employed to inactivate any genes that is not essential for viability.

### aphA-6

The gene *aphA-6* from *Acinetobacter baumannii* encodes the aminoglycoside 3′ phosphotransferase and confers resistance to kanamycin and other related aminoglycoside antibiotics (Shaw et al. 1993). Hence *aphA-6* was chosen as a selectable marker to express exogenous gene and modify specific chloroplast genes (Bateman and Purton 2000; Muto et al. 2009; Macedo-Osorio et al. 2018).

### Others

In addition to the two strategies that require the insertion of resistance genes to obtain resistance, genetic mutations in some chloroplast genomes were reported to confer resistance to certain antibiotics in *Chlamydomonas*. Some aminoglycoside antibiotics that act on the small subunit of the ribosome interfere with the functional assembly of the ribosome, such as spectinomycin and streptomycin. For *Chlamydomonas*, specific mutations in the 16S RNA (*rrnS*) gene confer resistance to spectinomycin or streptomycin, while mutations in the 23S RNA (*rrnL*) gene can confer resistance to erythromycin (Newman et al. 1990).

However, point mutations in ribosomal RNA genes (*rrnS* and *rrnL*) confer resistance by reducing the sensitivity of affected ribosomes to antibiotics. These point-mutated gene markers must be integrated into specific sites in the plastid genome, and the original gene should be replaced by sufficient copy numbers for resistance. Although these mutations provide a selectable marker for chloroplast transformation, there are caveats. A proportion of spontaneous mutations can occur in the host, leading to antibiotic resistance in antibiotic-containing environments. Single antibiotic screening is prone to false positives, so it is better to use a combination of antibiotics to facilitate the selection of true transformants.

## Development of markerless technologies is the future

Antibiotic resistance marker genes are often controversial in terms of ecological protection and food safety in genetically modified organisms, and the markerless technology provides a new strategy to address the concerns raised by antibiotic marker genes. In addition to some of the resistance markers mentioned above, some new biosafety markers are also applied in *Chlamydomonas* chloroplast genetic engineering (Table 4).

**Table 4:** Most common used selectable markers for *Chlamydomonas* chloroplast transformation

Marker	Functional description	Type	References
Resistance markers			
*aadA*	Spectinomycin or streptomycin resistance	Antibiotic	Goldschmidt-Clermont 1991
*AphA-6*	Kanamycin resistance	Antibiotic	Bateman and Purton 2000
*rrnS*/*rrnL* mutant	Spectinomycin or streptomycin/erythromycin	Antibiotic	Newman et al. 1990
Biosafety markers			
*atpB*/*rbcL*/*tscA*/*psbA*/*psbH*	Restore phototrophic growth	Phototrophy	Boynton et al. 1988; Goldschmidt-Clermont 1991; Michelet et al. 2011; Bertalan et al. 2015; Wannathong et al. 2016
*ARG9*	Restore arginine synthesis	Complement auxotrophies	Remacle et al. 2009
*ptxD*	Oxidation of phosphite to phosphate	Function-added	Sandoval-Vargas et al. 2018
*codA*	converts 5-fluorocytosine to toxic 5-fluorouracil	Negative selection	Jackson et al. 2022

### Restoration of photoautotrophic defects

**Fig. 2:**
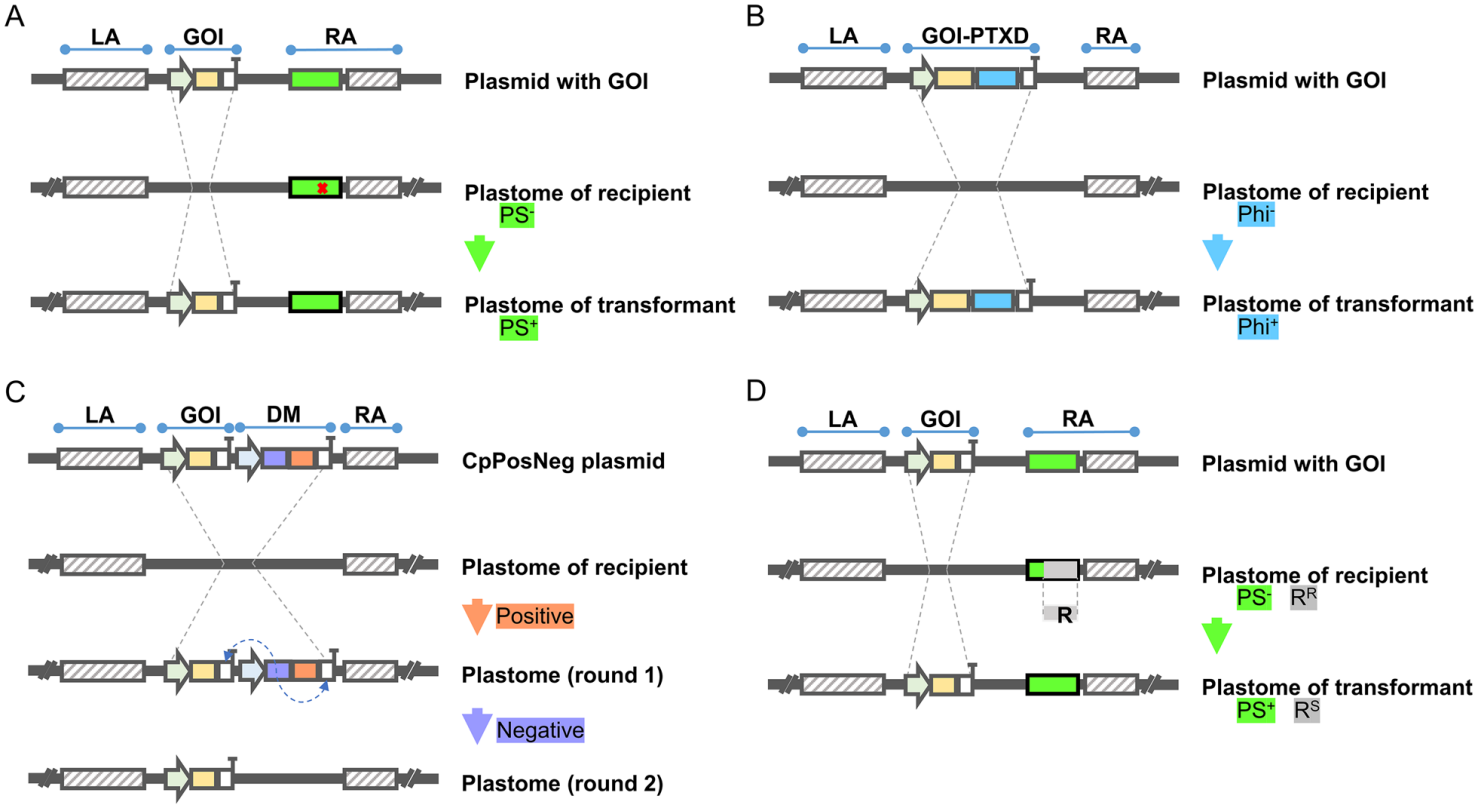
Schematic diagram of the marker-free transgenic strategy. *LA* left homologous arm; *GOI* gene of interest; *DM* dual marker; *RA* right homologous arm; *PS* photosynthesis; *PTXD* phosphite dehydrogenase; *Phi* phosphite; *R* resistance marker; **A** restoration of photoautotrophic defects. **B** Introduction of function-added selectable marker. **C** Propel antibiotic marker gene loss by negative pressure selection. **D** Homologous recombination of foreign genes into the genome to replace antibiotic marker gene

Under natural conditions, *C. reinhardtii* is photoautotrophic. In the absence of light, its growth depends on organic carbon sources, such as acetic acid. Several reports described various photosynthetic mutants that are mutated in essential photosynthesis genes (Doron et al. 2016; Shamriz and Ofoghi 2016; Esland et al. 2018). Deletion mutants of the chloroplast *atpB* gene were first used as photosynthesis-deficient hosts, allowing restoration of normal photosynthesis phenotype after bombardment with a wild-type DNA fragment spanning the deletion region (Boynton et al. 1988). This provides a powerful means to screen transformants, replacing the selection strategy of resistance gene markers (Fig. 2A). In addition, similar strategies were employed by other chloroplast photosynthetic genes, such as *rbcL* (Boynton et al. 1988), *tscA* (Goldschmidt-Clermont 1991), *psbA* (Michelet et al. 2011; Bertalan et al. 2015) and *psbH* (Wannathong et al. 2016). When the mutant-free photosynthetic metabolism gene was introduced as a homology arm into the corresponding deficient strain, the transformants recovered the photoautotrophic phenotype in minimal medium (without acetic acid).

### Construction of auxotrophs or function-added selectable marker

Complementary nutrition defective or additional special feature selectable markers could be utilized to screen the transgenic lines. The culture medium should be adjusted to confer certain stress conditions, such as the lack of essential nutrients. Once the completement gene is integrated into the chloroplast genome and encodes the functional enzyme, transformants have the ability to survive on the selective medium (Loppes and Heindricks 1986; Sandoval-Vargas et al. 2018, 2019; Changko et al. 2020).

For instance, the *ARG9* gene that located in *Arabidopsis thaliana* nucleic genome encodes N-acetyl ornithine aminotransferase, involving in arginine synthesis (Remacle et al. 2009). The auxotroph cells with the mutant *ARG* fail to grow in the arginine-containing medium (Loppes and Heindricks 1986). On account of the similarity between the codon bias of nuclear genome of *Arabidopsis* and the bias of *Chlamydomonas* chloroplast genome The *ARG9* cDNA of *A. thaliana* was successfully introduced into *Chlamydomonas* (*mt*^+^ *arg9-2* mutant) chloroplast genome. As expected, the auxotroph mutants were restored into arginine prototrophs. Thus, *A. thaliana ARG9* cassette has the potential to be developed as a screen marker for chloroplast transformation in *Chlamydomonas* (Remacle et al. 2009). Another example is the application of phosphite oxidation gene *ptxD* from *Pseudomonas stutzeri* WM 88, which encodes an NAD-dependent phosphite dehydrogenase that catalyzes the oxidation of phosphite to phosphate with the concomitant reduction of NAD^+^ to NADH (Costas et al. 2001). The *ptxD* gene was successfully inserted into the *Chlamydomonas* chloroplast genome and stably maintained in the plastome, accumulating the functional PTXD enzyme. The transplastomic lines could thrive in medium where phosphite is the sole source of phosphorus, whereas the proliferation of the wild type was inhibited (Sandoval-Vargas et al. 2018, 2019; Changko et al. 2020). This encouraging finding indicated that the *ptxD* gene can be utilized as a portable selection marker to replace antibiotic marker genes, and the phenotype is less prone to false positives. Cutolo et al. successfully expressed a chimeric endoglucanase (CelB)-PTXD protein in the *C. reinhardtii* chloroplast, avoiding insertion of antibiotic resistance marker genes into the plastomes (Cutolo et al. 2021) (Fig. 2B).

### Removal of antibiotic marker gene

The above two types of marker-free transgenic strategies make huge progress in the genetic engineering of *Chlamydomonas* chloroplasts, beyond them, the controversial resistance gene markers could also be removed by some ingenious methods. The latest study reported a rapid iterative marker system (CpPosNeg) for the transformation of *C. reinhardtii* chloroplasts from wild-type strains to marker-free transformants. This system employed a fusion protein consisting of a spectinomycin resistance protein and a cytosine deaminase as a dual marker. Transformants that carrying the spectinomycin marker were able to grow in the presence of the antibiotic in the first round of selection. The second round of selection was performed with 5-fluorocytosine, which promoted a rapid loss of the dual marker by intramolecular recombination between the 3′UTR of the marker gene and the 3′UTR of the transgene (Fig. 2C). Cytosine deaminase converts 5-fluorocytosine to toxic 5-fluorouracil, hence transformants that lost the fusion marker in the second round of selection survived, and only the foreign transgene was inserted into the wild-type chloroplast genome (Jackson et al. 2022). Another approach to remove resistance marker genes relies on complementation of photosynthetic defects. In Δ*psbH* strain (with downstream region deletion of the *psbH* gene), the *psbH* gene was inactivated by the insertion of the *aadA* gene. After the transforming plasmid (the entire downstream region of *psbH* as the homology arm) was introduced into the chloroplast of the Δ*psbH* strain, the *psbH* gene was restored and the *aadA* gene was replaced with the gene-of-interest cassette (Fig. 2D) (Wannathong et al. 2016). Thus, transformants were able to restore phototrophic growth on acetic acid-free medium and were sensitive to spectinomycin.

Recent study has demonstrated that the Cas9/gRNA-mediated gene editing system has been successfully used for gene editing in *Chlamydomonas* chloroplasts, enabling precise insertion of foreign gene (Yoo et al. 2020). This is also the first example that this system has been used for genome editing of *Chlamydomonas* chloroplasts. In this study the insertion of foreign genes was achieved by homology-directed repair (HDR) after Cas9 generated double-strand breaks. In addition, during the process of gene editing, the Cas9-expressing plasmid is not integrated into the plastome, avoiding unnecessary DNA substances other than foreign genes left in the genome after the editing plasmid is segregated and removed. In this way, precise gene editing can be achieved, and the antibiotic marker gene carried on the editing plasmid can be removed.

## Factors affecting expression efficiency need to be explored

### Transcriptional regulation

Promoter is the most fundamental component of the synthetic biology, driving the gene’s transcription. Rational promoter engineering can achieve predefined transcriptional control (Cazier and Blazeck 2021). In *E. coli*, in the absence of inducer, the products of negative regulator *lacI* gene form tetrameric repressor proteins that tightly binds to the operator downstream of the promoter, preventing the initiation of transcription. After the inducer (lactose, IPTG, etc.) is added, the inducer binds to the *lacI* gene product, which then dissociates from the operon and thereby activates transcription of the lac operon (Jacob and Monod 1961). Since the expression mechanism of chloroplast is similar to that of prokaryotes, Kato et al. inserted *lac* operating sequences into different locations of 16S rRNA and *rbcL* promoters, respectively, to evaluate their ability to induce transcription after the addition of inducer IPTG (Kato et al. 2007). As a result, the expression of reporter gene was control by the inducer at the transcriptional level. However, despite that this artificially controlled gene expression system is a suitable laboratory tool for analyzing the function of chloroplast genes, it is less suitable for large-scale expression of valuable products at industrial level due to the high cost of inducers and the difficulty of removing IPTG.

Since no known inducible promoter is known in *Chlamydomonas* chloroplasts, Rochaix et al. established a chloroplast inducible system using the trans-acting factor Nac2 (Rochaix et al. 2014). By introducing the chimeric *Cyc6* promoter-*Nac2* gene system into the *nac2* mutant, in the presence of copper, the *Cyc6* promoter could not drive *Nac2* expression, the chloroplast *psbD* transcript was unstable, and thus *psbD* expression was inhibited. If the exogenous gene GOI is fused with the 5′UTR of *psbD*, the expression of GOI can be achieved under copper-deficient conditions.

In other promoter engineering studies, exogenous proteins expressed in *C. reinhardtii* chloroplasts were mostly driven by endogenous promoters and 5′UTR (Barnes et al. 2005; Michelet et al. 2011; Rasala et al. 2011). High levels of mRNA accumulation were achieved using *atpA* and *psbD* promoters and 5′UTRs to drive the expression of exogenous gene *gfp* (Barnes et al. 2005). After fusion of the 16S rRNA promoter with *atpA* or *psbA* 5′UTR, the mRNA level of the exogenous gene *luxCt* was increased. Differently, for *atpA* 5′UTR, protein LuxCT was accumulated with the accumulation of mRNA, while for *psbA* 5′UTR, increased mRNA levels had little effect on protein accumulation (Rasala et al. 2011). In *C. reinhardtii* chloroplasts, distinct 5′UTRs led to significant differences in the mRNA and protein accumulation of exogenous genes, while 3′UTRs appeared to have relatively little effect.

### Post-transcriptional regulation

The transcript usually requires further processing, and mRNA binding proteins bind to the 5′UTR or 3′UTR of the transcripts, protecting the transcripts from degradation by exonucleases with 5′–3′exonuclease activity (Macedo-Osorio et al. 2021). The helical repeat protein family is a type of RNA-binding proteins involved in post-transcriptional gene expression, including the Half-a-tetratricopeptide repeat (HAT), Pentatricopeptide repeat (PPR) and Octotricopeptide repeat (OPR) proteins. Most of these trans-acting factors are encoded by the nuclear genome and are classified by their functions as mRNA maturation/stability (M) and translation (T) factor in post-transcriptional and translational processes, respectively (Cavaiuolo et al. 2017).

Three HAT proteins were experimentally characterized: NAC2 (alias MBD1) (Kuchka et al. 1989; Boudreau et al. 2000; Surzycki et al. 2007), MAC1 (Douchi et al. 2016) and MBB1 (Vaistij et al. 2000a, b; Loizeau et al. 2014). They bind to the 5′UTR of *psbD*, *psaC* and *psbB*, respectively, and resist degradation by the exonucleases. Moreover, MBB1 is involved in the processing of *psbB*–*psbT*–*psbH* polycistronic transcripts, and also binds to the 5′UTR of the smaller *psbH* transcript (Loizeau et al. 2014).

In *C. reinhardtii*, 14 PPR proteins were identified (Tourasse et al. 2013), but only 4 PPR was characterized as the M factor. MCA1 (PPR14) (Loiselay et al. 2008), MRL1 (PPR2) (Johnson et al. 2010) and TCB1 (PPR1) (Cavaiuolo et al. 2017) bound to the 5′-end of *petA*, *rbcL* and *petB* mRNAs, respectively protecting the transcripts from exonucleases degradation. PPR7 was shown to be a part of ribonucleoprotein complex that not only stabilized the *rbcL*, *rpoC2*, *psbH* and *tscA* transcripts, but also processed *atpA*–*psbI*–*cemA*–*atpH*, *psbJ*–*atpI*–*psaJ*–*rps12* and 16S rRNA operon polycistronic transcripts (Jalal et al. 2015).

Bioinformatic analysis of the *Chlamydomonas* genome revealed more than 120 OPR proteins that have not been fully experimentally confirmed (Macedo-Osorio et al. 2021). Previous studies showed that, similar to PPR and TPR, OPR proteins were extensively involved in the post-transcriptional control of chloroplast gene expression. As examples, MBC1 (OPR56) (Cavaiuolo et al. 2017), MBI1 (Wang et al. 2015), MCG1 (Wang et al. 2015), MCD1 (Murakami et al. 2005), MDA1 (Viola et al. 2019) and MTHI1 (Ozawa et al. 2020) act as M factors responsible for the stabilization of *psbC*, *psbI*, *petG*, *petD*, *atpA* and *atpH/I* mRNAs, respectively. Some OPR proteins participated in the RNA splicing process, including RAA1, RAA3, RAA8 (OPR120) and RAT2 jointly involved in the splicing of *psaA* mRNA (Reifschneider et al. 2017). However, spontaneous NCC1/NCC2 mutants in *C. reinhardtii* target the coding regions of two chloroplast transcripts, leading to destabilization of the *atpA*/*petA* transcripts, respectively (Boulouis et al. 2015).

Very recently, orthogonal regulators—those do not interact with host regulators—were first introduced into the *C. reinhardtii* to activate the expression of chloroplast transgenes (Shahar et al. 2021). The HCF107 protein (belong to HAT) in *Arabidopsis thaliana* is an orthologue of MBB1 in *C. reinhardtii* (Felder et al. 2001). Maize PPR10 activates expression of the chloroplast *atpH* gene at the post-transcriptional level (Pfalz et al. 2009; Prikryl et al. 2011). Tests indicated that ectopic expression of HCF107 and PPR10 in *C. reinhardtii* performed their native functions for *psbH* and *atpA* mRNA stability, respectively (Shahar et al. 2021).

The highly abundant accumulation of exogenous transgenic mRNA in chloroplast might be achieved by modifying the binding site of 5′UTR by relying on the post-transcriptional regulatory mechanism of natural nuclear coding proteins. In addition, the 3′UTR of *rbcL* is frequently used in chimeric structures downstream of heterologous genes, since the two inverted repeat sequence of the 3′UTR are *cis*-acting elements that regulate processing and stability of the transcript (Goldschmidt-Clermont et al. 2008).

### Translational regulation

Post-transcriptional processing and translation of mRNA occur simultaneously. Some OPR proteins play the function of T factor and participate in the translation process of mRNA, such as TAA1 (Lefebvre-Legendre et al. 2015; Reifschneider et al. 2017), TAB1 (Rahire et al. 2012), TBC2 (Auchincloss et al. 2002) and TDA1 (Eberhard et al. 2011; Carrera-Pacheco et al. 2020) involved in the translation of *psaA*, *psaB*, *psbC* and *atpA* mRNA respectively. Besides, different hosts have different codon preferences for gene expression. In *Chlamydomonas* chloroplasts, adenine (A) or thymine (T) is preferred at the third nucleotide of codon position (Nakamura et al. 2000). Absence of tRNAs matching the allogeneic codons in *Chlamydomonas* chloroplasts results in amino acid misincorporation and polypeptide chain truncation. One solution to this problem is to optimize the heterologous gene codons rationally, and to replace the rare codons of the foreign gene with more frequent codons in the expression host. *C. reinhardtii* chloroplast native tRNA^Trp^ is encoded by *trn*W_*UGG*_, which translates UGG codons to tryptophan. After the introduction of a temperature-sensitive *trn*W_*UCA*_ gene into the chloroplast genome, the stop codon UGA could be translated into tryptophan, achieving cold-induced translation of UGA codons (Young and Purton 2018). However, considering the high cost of cooling, such temperature-induced system seems difficult to be practically applied in industry.

### Post-translational regulation

Proteolysis is one of the key factors affecting protein expression. The N-terminal region of proteins is a major determinant of protein stability in bacteria and eukaryotes (Bouchnak and van Wijk 2019). The N-end rule pathway is a proteolytic system present in almost all organisms. The prokaryotic proteasome-like apparatus consists of ATPase modules (e.g., ClpA, ClpX) and covalently linked (e.g., Lon) or dispersed proteolytic modules (e.g., ClpP), mediating the degradation of misfolded proteins in the cytoplasmic matrix (Mogk et al. 2007; Tasaki et al. 2012). Chloroplasts of *C. reinhardtii* contain proteases commonly found in bacteria, such as Clp, Deg and FtsH proteases (Mayfield et al. 2007; Zou and Bozhkov 2021). Altering the N-terminal region of exogenous protein could increase protein abundance. If the transgene is fused to a small portion of the gene coding region at the N-terminus of the endogenous chloroplast protein rather than to its AUG translation initiation codon, the proteolysis can be alleviated and high levels of expression will be accumulated (Kasai et al. 2003; Michelet et al. 2011; Hsu et al. 2019). Furthermore, the chloroplast of *C. reinhardtii* is regarded an attractive platform for the expression of antigens or antibodies because of its ability to form the disulfide bonds required for the folding of these proteins (Mayfield et al. 2003; Taunt et al. 2018; Shamriz and Ofoghi 2019).

However, the chloroplast organelle lacks glycosylation modification pathways. The enzymes involved in glycosylation are located in the endoplasmic reticulum and the Golgi apparatus, and the nuclear expression productions of the transgenes will undergo glycosylation modification (Mathieu-Rivet et al. 2017). Reconstructing the glycosylation pathway in the chloroplast is impractical, and it is extremely challenging to export proteins expressed in the chloroplast to the cytoplasm for glycosylation. Considering about this limitation, chloroplasts are ideal for producing proteins with little or no glycosylation.

## Remaining challenges and future prospects

**Fig. 3:**
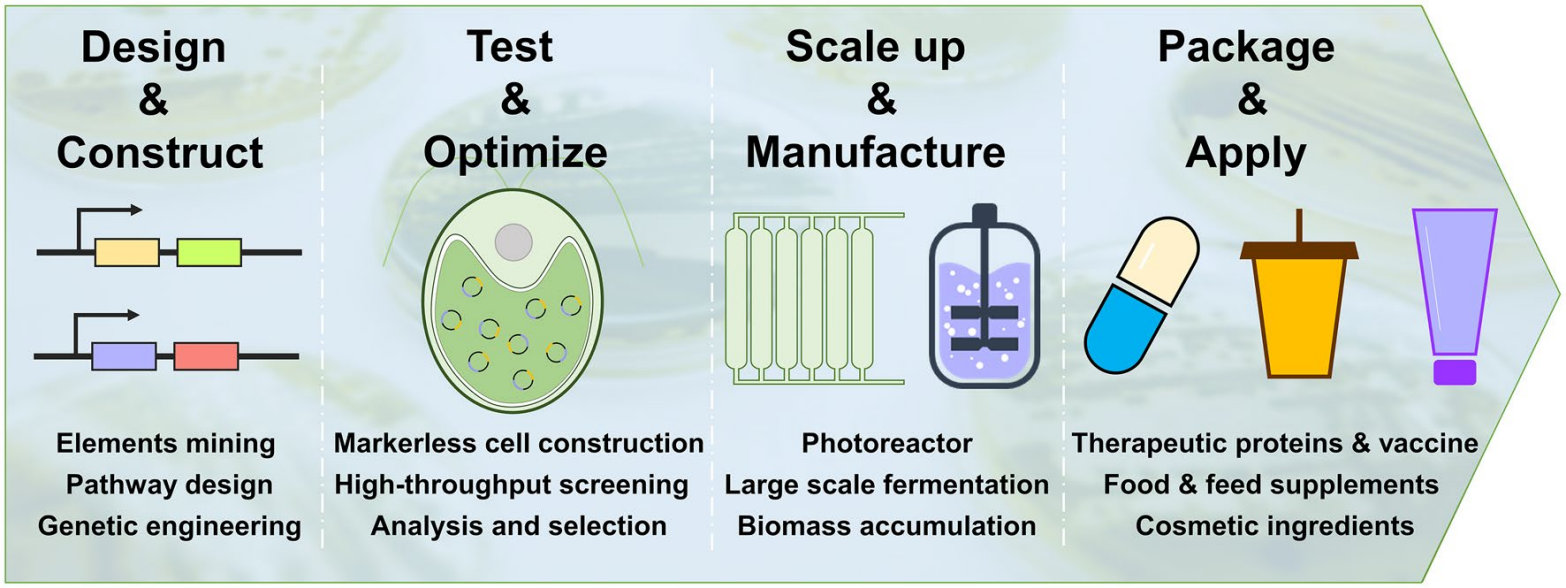
Rational design of *Chlamydomonas* chloroplast factories. The rationally designed expression vector is stably transformed into *Chlamydomonas* chloroplasts and achieved homoplasmy to construct transformants expressing the target protein. Large-scale culture of optimized transformants with excellent protein performance to produce high-value products for food, cosmetic and pharmaceutical industries

Although *Chlamydomonas reinhardtii* chloroplast has a prokaryotic expression mechanism, it contains a variety of molecular chaperones, protein disulfide isomerases and peptide isomerases that assist in the folding of complex proteins. This characteristic endows the *Chlamydomonas reinhardtii* chloroplast as an expression factory to produce high value-added products that difficult to express (Fig. 3). On the other hand, it should be noticed that a significant disadvantage of exogenous protein expression in the chloroplast genome is the inability to glycosylate the target protein, limiting the production of protein products that need to meet glycosylation requirements.

Antimicrobial resistance gene screening markers are commonly used in laboratories and fermenters, while neither antibiotics nor antimicrobial genes used for genetic transformation should be ingested by animals or released into the natural environment. It is essential to alleviate concerns about escape and horizontal transfer of antibiotic resistance markers. Therefore, it is necessary to develop novel, environment-friendly PTXD-like selection markers.

Analyzing the influencing factors of chloroplast gene expression is helpful to construct a novel and reliable exogenous protein expression platform. The limited number of endogenous promoters available is often a bottleneck in transcriptional regulation. Hybrid promoter engineering and the saturation mutagenesis of − 35 and − 10 motifs spacer regions are rational methods to modify prokaryotic promoter strength (Blazeck and Alper 2013), which is the first vital step to improve the activity of transcription for altering gene expression. With the analysis of a large number of promoter samples, the characteristics of promoter sequences can be summarized, and various structural parts of specific functions could be integrated together, and new promoter libraries of various strengths could be constructed.

The accumulation of endogenous proteins depends on the activity of multiple factors, which promote the stabilization and maturation of transcripts, further facilitate the translation of mRNA and enhance the stability of post-translational proteins. Therefore, it may be possible to assemble multiple M and T factors to achieve precise regulation of the processing and translation of foreign gene mRNAs. Moreover, drawing on the post-transcriptional regulation and expression mechanism of natural nuclear-encoded proteins, the binding site of the modified 5′UTR could be modified to recruit transcriptional or translational activators to achieve high-abundance accumulation of exogenous chloroplast transgenic mRNA or protein. Furthermore, constructing orthogonal regulatory systems can avoid host regulatory network disorder.

For the nuclear genome, Crozet et al. developed a Modular Cloning (MoClo) toolkit. This toolkit contains 7 promoters, 8 Immuno-purification tags, 12 UTRs (5′UTRs and 3′UTR), 12 reporters and 5 antibiotic resistance markers. It maximizes the modularity of expression elements for nuclear genetic engineering, which allows rapidly use of this toolkit for synthetic biology design in *Chlamydomonas* (Crozet et al. 2018). For the chloroplast genomes, similar toolkits can also be generated. In addition, by crossing, the chloroplast genome can be adapted to different nuclear genetic backgrounds. As long as the original chloroplast modification is in a *mt*^+^ strain (Joo et al. 2022).

With the rapid development of microalgal biotechnology, it is believed that chloroplasts with their unique advantages might become a low-cost and sustainable platform for large-scale production of valuable metabolites and compounds in the near future. Nevertheless, scale-up is one of the key steps to obtain these products on an industrial level. Only by solving the complex problems in large-scale research and improving the technical level can the chloroplast factory make progress in the production and manufacturing of the industrial level.
